# Transforming the future of ophthalmology: artificial intelligence and robotics’ breakthrough role in surgical and medical retina advances: a mini review

**DOI:** 10.3389/fmed.2024.1434241

**Published:** 2024-07-15

**Authors:** Eleftherios Chatzimichail, Nicolas Feltgen, Lorenzo Motta, Theo Empeslidis, Anastasios G. Konstas, Zisis Gatzioufas, Georgios D. Panos

**Affiliations:** ^1^Department of Ophthalmology, University Hospital of Basel, Basel, Switzerland; ^2^Department of Ophthalmology, School of Medicine, University of Padova, Padua, Italy; ^3^The Stoneygate Eye Hospital, Leicester, United Kingdom; ^4^Department of Ophthalmology, School of Medicine, Aristotle University of Thessaloniki, Thessaloniki, Greece; ^5^Department of Ophthalmology, Queen’s Medical Centre, Nottingham University Hospitals, Nottingham, United Kingdom; ^6^Division of Ophthalmology and Visual Sciences, School of Medicine, University of Nottingham, Nottingham, United Kingdom

**Keywords:** artificial intelligence, robotics, medical retina, surgical retina, vitreoretinal surgery, macular degeneration, retinal conditions, retinopathy of prematurity

## Abstract

Over the past decade, artificial intelligence (AI) and its subfields, deep learning and machine learning, have become integral parts of ophthalmology, particularly in the field of ophthalmic imaging. A diverse array of algorithms has emerged to facilitate the automated diagnosis of numerous medical and surgical retinal conditions. The development of these algorithms necessitates extensive training using large datasets of retinal images. This approach has demonstrated a promising impact, especially in increasing accuracy of diagnosis for unspecialized clinicians for various diseases and in the area of telemedicine, where access to ophthalmological care is restricted. In parallel, robotic technology has made significant inroads into the medical field, including ophthalmology. The vast majority of research in the field of robotic surgery has been focused on anterior segment and vitreoretinal surgery. These systems offer potential improvements in accuracy and address issues such as hand tremors. However, widespread adoption faces hurdles, including the substantial costs associated with these systems and the steep learning curve for surgeons. These challenges currently constrain the broader implementation of robotic surgical systems in ophthalmology. This mini review discusses the current research and challenges, underscoring the limited yet growing implementation of AI and robotic systems in the field of retinal conditions.

## Introduction

Artificial intelligence (AI) is a subfield of computer sciences with the goal of creating intelligent machines (1). Deep learning (DL), as a subfield of machine learning, allows computational models consisting of numerous layers, to learn representations of data characterized by varying degrees of abstraction (2). Ophthalmology is one of the specialties with widespread experimental implementation of AI. Deep learning (DL) can be extremely assistant in automated diagnosing of medical retinal diseases, like neovascular AMD, retinopathy of prematurity, diabetic retinopathy as well as of surgical retinal diseases, such as macular hole, retinal detachment and epiretinal membrane. On the other side, the emerging advancement of robotics has led to the introduction of robotics into a variety of surgical medical specialties like gynecology, urology and general surgery (3). Eye surgery necessitates sufficient lighting, stabilization of the hand movements and unobstructed view (4). Robotic surgical systems offer specialized features that can effectively fulfill these requirements, ensuring optimal conditions for surgical precision and success.

Considering the above, the primary aim of this review is to raise awareness about the novel applications of artificial intelligence and robotic technology on the management of vitreoretinal diseases. Recent publications on the field of AI and deep learning application in the diagnosis of medical and surgical retina disorders along with the challenges and limitations are going to be summarized. Moreover, published data on novel robotical approaches in the surgical management of retinal disorders are going to be analyzed.

## Methods

For this mini-review, we conducted a search in PubMed, Embase and Scopus using the keywords “(Automated diagnosis) AND (OCT* OR Optical Coherence Tomography*),” “(‘Artificial Intelligence’ OR ‘Machine Learning’) AND ‘Retina’ ‘and’ (Robotics) AND (Ophthalmology).” These searches were performed between 27 February 2024, and 1 April 2024. To be included in the review, studies needed to focus on either the automated diagnosis of OCT images using deep learning algorithms for surgical or medical retinal diseases, or the application of robotics in ophthalmology. Studies without English manuscript or clinical relevance were excluded.

## Automated diagnosis through the utilization of machine learning and deep learning algorithms

Convolutional neural network (CNN) is a deep learning model designed to analyze data exhibiting a grid-like structure, such as images (5). Modern architecture CNNs are employed to perform the segmentation of the retinal OCT. The development of an algorithm necessitates a large dataset of fundus images (OCT, fundus or sonography images) as a reference for the system to differentiate the anatomical structures on the images such as retinal layers or pathological abnormalities. In OCT images, segmentation and masking of retinal layers are conducted to emphasize the region of interest, while excluding extraneous background regions (6). Fundus images are being processed on a gray-scale mode where the pixel values of the background equated to zero, while the pixel value of the active area is above zero (7).

In general, the performance of these algorithms is evaluated in most published studies using metrics such as AUC (area under the ROC curve), specificity, sensitivity, accuracy, intraclass coefficients, or by comparing them to ophthalmic specialists or residents for accuracy in a series of clinical cases.

## Artificial intelligence and deep learning in color fundus photography and optical coherence tomography (OCT)

One of the first applications of AI and Dl in retinal diseases, was the automated interpretation of color fundus photos for the screening of age-related macular degeneration (AMD) through the study of Van Grinsven et al. (8), who presented a system for differentiating high-risk from low-risk patients for non advanced AMD. The authors reported that the detection of drusen was highly similar between the human observers and the machine learning system with a mean intraclass correlation coefficient (ICC) over 0.85. In terms of risk prediction for AMD the proposed system reached the receiver operating characteristic (ROC) curve of 0.948 and 0.954, achieving similar performance with the human observers.

In the field of ROP, a prompt and accurate diagnosis is of paramount importance. ROP is also an area of ophthalmology that AI has been widely implemented over the last few years. Brown et al. (9) were the one of the first to create and validate a deep convolutional neural network for the automated diagnosis of plus disease in ROP with the use of 5,511 retinal images from the cohort study imaging and informatics in retinopathy of prematurity (i-ROP). The authors reported a 93% sensitivity and 94% specificity for identification of plus disease, achieving comparable or better accuracy than human retinal experts (9).

A deep convolutional neural network proposed by Li and Liu was trained for the diagnosis of ROP for stages I, II and III, using 18,827 fundus images from premature infants. This proposed network achieved a high specificity and sensitivity for all three stages of ROP (Table 1) with the highest an average AUC score of 0.9663 (10). More recently, another computer algorithm was published from Sharafi et al. (11) for the automated diagnosis of plus disease in ROP through accurate segmentation of retinal vessels.

**TABLE 1:** Overview of studies with application of novel DL-algorithms in medical retina diseases.

Disease	References	Number of images used for the development of the algorithm	Key findings
ROP	Brown et al. (9)	5,511 retinal images	• For plus disease 93 % sensitivity with 94% specificity • For pre–plus disease or worse, 100% sensitivity and 94% specificity were 100% • Quadratic-weighted κ coefficient of 0.92
	Li and Liu (10)	18,827 retinal images	• ROP Stage I: 90.21% sensitivity with 97.67% specificity • ROP Stage II: 92.75% sensitivity with 98.74% specificity • ROP Stage III: Sensitivity of 91.84% with 99.29% sensitivity • For normal images, 95.93% sensitivity and 96.41%, specificity
	Sharafi et al. (11)	76 retinal images	• Accuracy in differentiating between Plus and non-Plus images, 0.86 ± 0.01
AMD	Van Grinsven et al. (8)	407 color fundus images	• For automatic AMD risk assessment: (ROC) curve of 0.948 and 0.954
nAMD	Jang et al. (12)	888 Cirrus and 556 Spectralis OCT images	• AUC of 0.725 ± 0.012 for the model with the fluid region of OCT following the loading phase
dAMD	Pramil et al. (13)	126 en face swept-source OCT images	• 0.99 ICCs o for the GA measurement and 0.94 ICCs for the progression rates of GA area, respectively.
Myopic maculopathy	Ye et al. (14)	2,342 OCT images	• For all 5 different entities of myopic maculopathy AUC from 0.927 to 0.974 • Sensitivity of 56.16–99.73% equal to or better than those of junior retinal practicians.
Central serous chorioretinopathy	Aoyama et al. (17)	100 OCT-A en face images	• Mean accuracy rate of 88% for the Keras-Ten- sorflow and 95% for Neural Network Console. • Not significant difference (*P* > 0.01) between the two models.
	Ko et al. (16)	7425 Spectral Domain-OCT images	• Average cross-validation accuracy of 94.2% (95% CI 0.897–0.986), with sensitivity of 94.9% and specificity of 99.1%. • Equal to superior performance compared to other CNN-based models as well as compared to ophthalmic specialists.

AUC, area under the ROC curve; AMD, age-related macular degeneration; dAMD, dry age-related Macular Degeneration; nAMD, neovascular age-related macular degeneration; CNN, convolutional neural network; GA, geographical atrophy; ICC, intraclass correlation coefficient; OCT, optical coherence tomography; OCT-A, optical coherence tomography angiography; ROP, retinopathy of prematurity; ROC, receiver operating characteristic curve.

In the spectrum of nAMD, Jang et al. (12) recently designed a deep learning algorithm for the prediction of the first recurrence following three loading injections of anti-VEGF. This study included 1,444 eyes from 1,302 patients. Their findings illustrate that a model utilizing OCT scans of the fluid region post-loading phase exhibited the top classification performance, achieving an area under the receiver operating characteristic (AUC) of 72.5%. Analysis via heatmap uncovered that three distinct pathological fluids (PED, SRF, IRF), diminished choroidal neovascularization lesions and hyperreflective foci were significant regions associated with the initial recurrence.

Automated algorithms have been also introduced for dry AMD. One of them from Pramil et al. (13) employed for the training of the algorithm using 126 SST-OCT images scans from 90 individuals with geographical atrophy, 32 individuals with early or intermediate AMD and 16 healthy controls. For the development of the algorithm 3 parameters were used for the automated segmentation: hypertransmission within the subretinal pigment epithelium layer, areas exhibiting RPE absence, and reductions in retinal thickness. The algorithm demonstrated strong consistency, achieving ICCs (intraclass correlation coefficients) of 0.99 and 0.94 for the measurements of geographic atrophy (GA) area and the rates of GA area enlargement, respectively.

Regarding myopic maculopathy, several DL-based models have been proposed that aid for an automated diagnosis based on the OCT-images. Ye et al. (14) introduced an algorithm that was developed with the use of more than 2,300 OCT Spectralis HRA (Heidelberg Engineering, Heidelberg, Germany) images and was developed for the detection of five entities of the myopic maculopathy: macular choroidal thinning, macular Bruch membrane (BM) defects, subretinal hyper-reflective material (SHRM), myopic traction maculopathy (MTM), and dome-shaped macula (DSM). This neural network recorded an AUC from 0.927 to 0.974 for the different entities of myopic maculopathy as well as equal to better sensitivities than junior specialists.

Central serous chorioretinopathy (CSC) is distinguished through localized serous detachment of the macula and affects mostly young males (15). The diagnosis of CSC has relied on invasive imaging techniques, such as fluorescein angiography (FA) and indocyanine green angiography (ICGA). Deep learning finds application in the diagnosis of CSC, with a proposed algorithm that included 7425 SD-OCT images from 297 individuals (16). This diagnostic system achieved an average cross-validation accuracy of 94.2% (95% CI 0.897–0.986), with a sensitivity of 94.9% and specificity of 99.1%. Similarly, another DL algorithm have shown sufficient efficacy in the automated diagnosis of CSC (17).

## Surgical diseases

With regard to surgical retinal diseases, a plethora of algorithms based on DL have been published for automated diagnosis of clinical entities such as epiretinal membrane, macular hole and retinal detachment. To start with, few studies have been focused on epiretinal membranes; Lo et al. (18), who used 3,141 OCT images, introduced an algorithm with an AUC of 0.99, sensitivity 0.99 and specified 0.98. More recently, Tang et al. (19) introduced a model using 468 OCT images, which helped junior physicians significantly to increase their accuracy at diagnosing epiretinal membrane.

With relation to retinal detachment, Wang et al. (20) introduced and validated an DL-algorithm in China using 6,000 ophthalmic ultrasound images from 1,645 participants. The top-performing DL model in detecting RRD in this study attained an area under the ROC curve (AUC) of 0.998, accompanied by a sensitivity of 99.2% and a specificity of 99.8%. This DL model could be extremely helpful on the countryside, where there is no wide availability of ophthalmologists. In addition, Fung et al. (21) trained a DL model for the prediction of postoperative anatomical outcomes after RRD surgery. The authors used 6661 digitally drawn RRD images from patients that had undergone pars plana vitrectomy with endotamponade in order to train the DL model. This model achieved an AUC of 0.94, with sensitivity of 0.73 and specificity of 0.96.

DL algorithms have also been developed for the diagnosis of RDD based on fundus images (Table 2). Li et al. (22) trained an algorithm with 24.208 ultra-widefield fundus images for the localisation of the anatomical retinal detachment areas. The authors used various localization systems in the development of the algorithm, which recorded an 86.42% precision and an 83.27% recall in the 48-partition lesion detection and a 92.67% precision with a recall of 68.07% in the baseline model. The algorithm for holistic lesion localization achieved 89.16% precision and 83.38% recall.

**TABLE 2:** Overview of studies with application of novel DL-algorithms in surgical retina diseases.

Disease	References	Number of images used for the development of the algorithm	Key Findings
Epiretinal membrane	Lo et al. (18)	3,618 OCT images	• 98.7%, specificity: 98.0%, and F1 score: 0.945. • AUC of the ROC curve was 0.999. • Slightly better score than non-retinal specialized ophthalmologists in the diagnosis of ERM.
	Tang et al. (19)	468 OCT images	• Image-level accuracy of 95.65%, and ERM region-level accuracy of 90.14% • Significant improvement of the accuracy of the clinicians in detecting ERM.
Retinal detachment	Fung et al. (21)	6,661 retinal images	• AUC of 0.94, sensitivity of 73.3% and a specificity of 96%
	Wang et al. (20)	5,000 ultrasound images	• The most effective DL model achieved an AUC of 0.998, coupled with a sensitivity of 99.2% and specificity of 99.8%.
	Li et al. (22)	24,208 ultra-wildfield images	• 48-partition lesion detection: 86.42% precision and 83.27% recall with an average precision of 0.9132. •B aseline model: 92.67% precision and recall of 68.07% •H olistic lesion localization: 89.16% precision and 83.38% recall.
Macular hole	Valentim et al. (23)	601 OCT images	•T est set 1: Sensitivity 75.7%, Specificity 94.8%, Accuracy 88.5%, AUC 90.2% • Test set 2: Sensitivity 89.5%, Specificity 94.2% Accuracy 91.4%, AUC 95.5%

AUC, area under the ROC curve; DL, deep learning; ICC, intraclass correlation coefficient; OCT, optical coherence tomography; ROC, receiver operating characteristic curve.

Another DL-based model has been introduced from Valentim et al. (23) for the automated detection of idiopathic full thickness macular holes with the use of spectral domain optical coherence tomography. More than 600 cube scans from patients were used to train the algorithm. An accuracy of 88.5% was mentioned by the authors together with the need for further training of the algorithm in order to recognize the different stages of macular holes.

## Robotic Surgery

In this section, a comprehensive synopsis of the most important clinical studies on application of robotics in vitreoretinal diseases along with an overview of the most prominent surgical robotic systems in ophthalmic surgery is provided.

Ophthalmic surgery is a surgical field that demands a high level of precision and accuracy in a series of sensitive tissue manipulations that can last up to hours. For example, from anatomical point of view, an epiretinal membrane has a mean thickness of ca. 60 microns, necessitating high precision during removal to avoid injury to nearby tissues. Moreover, some factors like hand tremor, excessive number of tissue manipulations and surgeon’s fatigue can have a negative impact on the surgery. Hence, robotic technology offers a variety of advantages in the operative theater. Firstly, robotic technology can achieve a precision of 1 mm (3). Furthermore, robotic surgical systems can reduce the hand tremor, whilst offering a 360° range of movement intraoperatively. The accuracy of robotic systems can be further warranted through the incorporation of intraoperative OCT, allowing for better intraoperative visualization, like already mentioned above.

The most prominent robotic surgical systems in ophthalmology is the da Vinci system (Intuitive Surgical, USA) with more than 4,000 unit installations worldwide until 2017 (24). It is composed from two primary components, a control console and the robotic apparatus. Da Vinci system offers optical magnification of the surgical field together with the feature of reducing the hand tremor of the surgeon. Another benefit of this surgical system is the 360° range of motion of the joints, enabling optimal positioning and enhancing precision during the ophthalmic surgery (3).

The Intraocular Robotic Interventional Surgical System (IRISS) is a contemporary telemanipulated robotic surgical system that was developed for reducing complication rates in cataract extraction (25, 26). IRISS possesses an integrated OCT and a stereo-camera that allows for preoperative planning and accurate intraoperative intervention. IRISS consists of two surgical manipulating arms that can simultaneously be remotely controlled through a distant operator. T his surgical system is capable of simultaneously attaching up to four tools and seamlessly transitioning between them during surgical procedures.

Preceyes surgical system (PSS, Preceyes bv, Eindhoven, the Netherlands) is another robotic surgical system that has been designed for surgical procedures of the posterior segment, such as vitrectomy or subretinal injections. It carries the advantage of uncomplicated integration into the operating microscope while the surgeon could be positioned at his usual surgical site. PSS offers the ability to incorporate useful surgical instruments, such as forceps, scissors, and cutters that can be easily operated through a foot pedal (2). This robotic surgical system offers a significant intraocular reach spanning of 130 degrees from a temporal opening (27).

In a prospective randomized clinical study from Faridpooya et al. (28), 15 patients with idiopathic ERM after randomization received either a manual surgery or a robotic-assisted one, with the aid of the Preceyes surgical system (PSS, Preceyes bv, Eindhoven, the Netherlands). Results showed no complications in the PSS group, as well as shorter distance traveled by the forceps. With regard to visual outcomes, similar results were reported between the two groups, with longer mean surgical time in the PSS group (PSS: 56 min vs. Manual Surgery: 24 min). Moreover, a comparative study was undertaken to assess the efficacy of robotic versus manual peeling techniques (3). Initially, manual peeling was performed with the aid of Eyesi surgical simulator (VRmagic, Mannheim, Germany) followed by the application of the Preceyes surgical system from the same surgeon. The findings demonstrated that manual peeling took 5 min on average, whereas peeling assisted by the robotic system required an average of 9 min. Additionally, the robotic approach necessitated an average of 344 mm to complete the ILM peeling, whereas the manual method utilized approximately 600 mm. Overall, the robot-assisted procedure was found to be safer due to reduced number of macular hemorrhages during the peeling and because of total eliminated retinal injuries caused from the surgical instruments.

## Limitations, challenges and future aspects

Robotic eye surgery, while a frontier of innovation, faces substantial challenges that could impede its broader adoption. The complexity of mastering robotic systems presents a significant learning curve, which may deter many surgeons from transitioning to these technologies. The high costs associated with purchasing, maintaining, and training personnel on robotic systems pose financial burdens that many institutions may find prohibitive. The time-intensive setup and the need for specialized training can limit the practicality of robotic systems in high-throughput surgical environments. Furthermore, issues such as suboptimal endoscopic visualization, which lacks the detail provided by traditional microscopes, and the potential for increased ocular surface stress due to the remote center of motion, further complicate the use of systems like the da Vinci robotic operating system (4).

Looking to the future, developments should focus on integrating robotic systems seamlessly into the existing surgical workflows, enhancing their adaptability to various surgical scenarios. Innovations in visualization technologies could mitigate current limitations, offering clearer, more detailed intraoperative views that could improve surgical precision and outcomes. Making robotic systems more cost-effective and easier to maintain could facilitate their adoption in a wider range of medical settings, potentially making them a standard tool in ophthalmic surgeries. Advanced simulations and training programs could be developed to reduce the learning curve and enhance the surgical skills necessary to operate these sophisticated systems effectively. Leveraging AI to integrate real-time data analytics during surgery could enhance decision-making processes and surgical precision, leading to better patient outcomes.

In the field of AI in ophthalmology, the journey is just beginning. As algorithms evolve, there is a continuous need for refinement and training of algorithms with larger, more diverse data sets, including complex and rare conditions, to enhance accuracy and reliability. Developing standardized protocols for AI applications in medical settings, addressing privacy, and ethical concerns related to patient data use are critical. Strengthening collaborations across disciplines—combining insights from data scientists, clinicians, and engineers—can drive innovations that fully harness AI’s potential in ophthalmology.

The integration of large language models (LLMs) like ChatGPT into ophthalmology offers promising advancements (29). These AI systems could improve diagnostics and patient education through real-time data analysis and access to vast research resources. LLMs have the potential to enhance decision-making and extend specialized care to underserved regions. However, their clinical application will require thorough validation and careful consideration of data security and ethical concerns to fully realize their transformative impact on ophthalmology.

These initiatives will not only overcome existing barriers but also expand the capabilities of AI and robotics in ophthalmology, setting a new standard for precision and efficiency in patient care.

## Conclusion

AI holds tremendous promise as an emerging field in ophthalmology. To date, numerous deep learning-based algorithms have been developed for automated diagnosis of a plethora of medical and surgical retinal diseases. Yet, international collaborations remain imperative, alongside enhancing the capabilities of existing algorithms through extensive training with larger datasets encompassing complex cases. This approach aims to refine and optimize these algorithms for enhanced efficacy.

Additionally, robotic surgery presents a promising frontier in ophthalmology. However, it necessitates further research, particularly focused on pioneering robotic systems seamlessly integrated into the routine operations of ophthalmic theaters. Such advancements hold the potential to revolutionize surgical procedures, ultimately benefiting patients and practitioners alike.

## Author contributions

EC: Data curation, Formal analysis, Methodology, Resources, Writing – original draft. NF: Validation, Writing – review & editing. LM: Writing – review & editing. TE: Writing – review & editing. AK: Writing – review & editing. ZG: Writing – review & editing. GP: Conceptualization, Supervision, Validation, Visualization, Writing – original draft, Writing – review & editing.
